# Growth Targets Management, Regional Competition and Urban Land Green Use Efficiency According to Evidence from China

**DOI:** 10.3390/ijerph19106250

**Published:** 2022-05-20

**Authors:** Zhangsheng Liu, Binbin Lai, Shuangyin Wu, Xiaotian Liu, Qunhong Liu, Kun Ge

**Affiliations:** 1 Department of Engineering Management and Real Estate, College of City Construction, Jiangxi Normal University, Nanchang 330022, China; xiaoliu8033@jxnu.edu.cn (Z.L.); 202040100796@jxnu.edu.cn (B.L.); 202040100792@jxnu.edu.cn (S.W.); 402017010873@jxnu.edu.cn (X.L.); 001953@jxnu.edu.cn (Q.L.); 2 Institute of Real Estate, College of City Construction, Jiangxi Normal University, Nanchang 330022, China

**Keywords:** growth targets management, regional competition, urban land green use efficiency, super efficiency slack-based model, China

## Abstract

Based on the panel data of 257 prefecture-level cities in China from 2010 to 2017, this paper measured urban land green use efficiency (ULGUE), incorporating undesirable outputs, via the super efficiency slack-based model (SBM). It also explored the effect, mechanism, and heterogeneity of growth targets management and regional competition on ULGUE via the time-varying gravitational spatial weight matrix and the spatial self-lagging model. The results show that growth targets management and regional competition have significant positive effects on ULGUE, and enhance the ULGUE by promoting local investment attraction, increasing innovation inputs, optimizing environmental regulations and strengthening commercial activities. Additionally, growth targets management has a more significant effect on eastern cities, non-central cities, and mature urban agglomeration, while regional competition has a more significant effect on central cities, non-central cities, and developmental urban agglomeration. Therefore, considering development as the priority, setting relatively aggressive economic growth targets and optimizing the regional competition mechanism for growth targets management can help improve the ULGUE and promote high-quality economic development in China.

## 1. Introduction

Since China’s reform and opening-up, the urbanization rate of China has increased from 17.9% in 1978 to 63.89% in 2020, implying that the urbanization level has developed rapidly. However, existing studies have found that the problem of urban land use efficiency is a concern, and that sustainable socio-economic development faces serious challenges [1,2]. The reason is that urban land continues to expand excessively. This problem is accompanied by the increase of high energy consumption, high carbon emissions, and high pollution in urban land-use [3]. The rapid expansion of construction land is an unavoidable problem for economic growth, and regional competition has accelerated the spread of this phenomenon. British economist David Pearce first proposed the concept of “green development” in his work [4]. and his thinking resembles China’s “sustainable development” mode. The idea of “green development” in China was formally put forward at the Fifth Plenary Session of the 18th Central Committee of the Communist Party of China [5]. Its essence is to minimize resource consumption and pollution emissions without reducing output. Given this context, the only way to achieve sustainable development is to improve land-use efficiency which would directly improve the ULGUE under environmental constraints. In China’s economic system, growth targets management centered around economic growth plays a crucial role in the economic and social fields [6]. What impact do they have on the ULGUE, and how does regional competition affect this? Some have proposed that economic growth targets should be suppressed [7,8], while others have affirmed the leading role of economic growth targets [9]. Unfortunately, the existing literature is not unanimous on this topic, which seriously confuses decision makers. For this reason, clarifying the impact of growth targets management and regional competition on the ULGUE and its mechanism has essential practical value and theoretical significance for China’s sustainable development and high-quality economic growth.

Reviewing the existing literature, most relevant studies have paid significant attention to improving urban land-use efficiency, focusing on its measurement and evaluation [10,11], spatial-temporal differences [3,12] and influencing factors [13,14]. As for the relevant research on growth targets management, the existing literature has explored the impact of economic growth targets on the economy [15], finance [16], innovation [17], etc. Nevertheless, research on the relationship between growth targets management and ULGUE is still lacking, and the internal mechanism needs to be improved. Furthermore, the New Economic Geography has shown that everything is spatially connected, and each regional variable has spillover effects on neighboring areas. Growth targets management is no exception. However, few studies consider the impact of regional competition based on spatial measurement methods. This makes it difficult to fully interpret the impact of growth targets management on the ULGUE.

To fill the research gaps mentioned above, this paper attempts to make possible marginal contributions from the following aspects: (1) based on China’s unique economic and political system, this paper systematically dissects the theoretical mechanism of the impact of growth target management and regional competition on ULUGE, filling the gaps in existing research; (2) the relationship between spatial units is considered in this paper, incorporating the regional competition in growth targets management into the research framework by constructing a time-varying gravitational spatial weight matrix, and the spatial self-lagging model is applied to empirically investigate the impact and mechanism of growth targets management and regional competition on the ULGUE in 257 prefecture level cities; (3) the differentiated effects of growth targets management and regional competition on ULGUE are analyzed in depth from the perspective of multidimensional heterogeneity, with a view to providing a realistic basis for enhancing ULGUE.

## 2. Theoretical Mechanism Analysis

**Figure 1:**
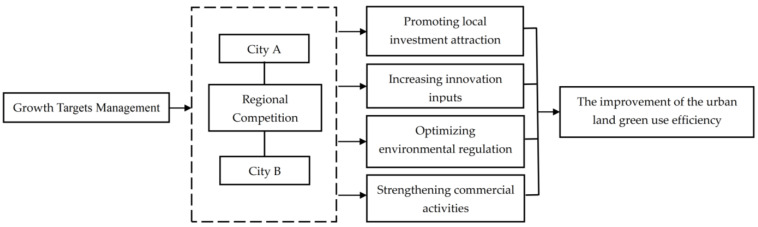
Analysis of the theoretical mechanism.

Under the political assessment mechanism centered on GDP, local authorities are paying more and more attention to economic growth. Local officials do not shy away from using resources and power to achieve short term economic growth and the resulting promotion [18]. At the same time, local government has significant horizontal competition when formulating economic growth targets, which has promoted the formation of regional competition in growth targets management [19,20]. The promotion model based on GDP evaluation is an essential means for central government to increase officials’ motivation. The existence of growth targets management also has a significant positive impact on actual economic growth [21]. Through literature review and logical analysis, this paper describes the channels through which growth targets management and its regional competition affect the ULGUE from the following four perspectives (as shown in Figure 1).

Firstly, growth targets management and regional competition will improve the ULGUE by promoting local investment attraction.

Foreign direct investment provides a solid financial foundation for improving ULGUE. If a region has a strong ability to attract foreign investment, solving problems such as efficient and green land is easy. This is particularly important because of the financial implications of this issue. Under the influence of growth targets management, local officials tend to set higher economic growth targets. To achieve these targets, they actively use resources and power to gain a large inflow of foreign direct investment. The empirical analysis results of Shen et al., (2021) [17] and Huang et al., (2021) [18] showed that setting economic growth targets significantly affects foreign direct investment. On the one hand, raising economic growth targets will significantly increase fiscal expenditures [22], thereby enhancing the region’s ability to attract foreign direct investment [16], improving the ULGUE. On the other hand, government increases output by increasing the land transfer area to achieve economic growth targets [23]. This promotes foreign investment to a certain extent and achieves a large inflow of foreign capital, which directly promotes the ULGUE [18].

Secondly, growth targets management and competition will improve the ULGUE by increasing innovation inputs.

The “green development” concept is an inevitable step in achieving sustainable urban development under resource and environmental constraints. It is also an essential guiding ideology and the primary way to achieve China’s comprehensive socio-economic transformation [24]. Improving the ULGUE requires innovative land technology, and transformation and enhancement of the governance mode and capabilities related to land resources. Hopefully, local government will invest more in scientific and technological innovation, considering the financial constraints that innovation faces. Studies have shown that China’s construction land development is rising, and investment in scientific and technological innovation has a significant role in promoting land intensive use [25]. Financial development can promote energy and environmentally biased technical progress. However, the impact of financial development on biased technical progress is heterogeneous due to the U-shaped relationship between economic development and resource endowment. The impact of energy and environmentally biased technical progress is also increasing [26]. Therefore, growth targets management urges local governments to invest more into regional scientific and technological innovation, further enabling them to achieve their economic growth target.

Thirdly, growth targets management and regional competition will enhance the ULGUE by optimizing environmental regulation.

The existing discussion on land-use efficiency has led to rich academic achievements, but mainly focuses on the construction of the evaluation index t the use level [27]. Thanks to the concept of green development, the relationship between environmental regulation and the ULGUE has also attracted extensive attention from scholars. The implementation of environmental regulation reduces the degree of environmental pollution by restricting the environmental actors. This implementation also promotes the green development of the environment and improves the ULGUE. For example, Zhang et al., (2021) found a significant double threshold relationship between environmental regulation and the ULGUE, using the micro-transmission mechanism. With appropriate intensity, environmental regulation can improve the ULGUE [28]. Song et al., (2018) found that the current economic growth can stimulate environment-oriented technological progress to improve the ULGUE [29]. Suppose that local governments want to achieve high economic growth targets and promote rapid economic development; then they must practice the green development concept known as “Lucid Waters and Lush Mountains are Invaluable Assets”. Many studies have shown that strict environmental regulation policies can facilitate the win–win of economic growth and environmental protection, at a relatively low level of environmental pollution [30]. Therefore, moderate management of economic growth targets will encourage regional green technology innovation through introduction of foreign capital and industrial structure, which will help boost the green development of cities [17]. Raising the economic growth targets will urge local governments to optimize environmental regulation.

Lastly, growth targets management and regional competition will improve the ULGUE by strengthening commercial activities.

Existing studies have shown that the impact of China’s commercial circulation industry on green ecological consumption is in line with the “inverted U-shaped” law. At present, the development of China’s commercial circulation industry has not reached the inflection point level. Strengthening commercial circulation activities can promote China’s green development, conducive to improving the ULGUE. The commercial circulation industry is a pillar of national economic development, and its activities are an important driving force for regional economic growth. For example, the development level of modern commerce and commercial circulation positively relates to the agricultural economic growth of coastal provinces and cities in China. With the continuous improvement of the development level of modern commerce and commercial circulation, these provinces and cities experienced an increase in agricultural economic development [31]. With the continuous expansion of the scale of China’s circulation industry, commodity circulation efficiency has become the most crucial factor in developing national competitiveness. The development efficiency of the circulation industry varies significantly in different regions, and the factors affecting it play different roles in different areas [32]. Toevska-Trpevska and Tevdovski (2016) found that commercial facilitation significantly promotes regional economic growth [33]. Therefore, growth targets management urges local governments to vigorously develop the commercial circulation industry and strengthen regional commercial activities to achieve high economic growth targets.

## 3. Method and Data

### 3.1. Model Specification

#### 3.1.1. Benchmark Regression Model

For the purpose of investigating the effects of growth targets management and regional competition on the ULGUE, this paper constructed a spatial self-lagging model based on the time-varying gravitational spatial weight matrix for analysis referring to Vega et al., (2015) [34]. This model has several advantages compared to the auto-regression and spatial Durbin models. First, it can directly estimate the direct and indirect effects of growth targets management and regional competition. Second, the estimation method is relatively simple and inclusive of the panel model, and the ordinary panel model estimation methods are also applicable. Third, the time-varying gravitational spatial weight matrix based on the gravity model enables a better interpretation of the dynamic evolution of the regional competition growth in targets management.

This paper constructed the following benchmark regression model:

(1) $$ULGUE_{it}=\alpha +\alpha_{1}gt_{it}+\alpha_{2}w_{t}\cdot gt_{it}+\gamma_{1}control_{it}+\mu_{i}+\varepsilon_{\mathrm{it}}$$

In this formula, i represents the city and t represents the year, while ULGUE is the explained variable. ${\mathrm{gt}}_{it}$ indicates the explanatory variable, growth targets management, and $w_{t}\cdot {\mathrm{gt}}_{it}$ represents another explanatory variable, the regional competition for growth targets management. ${\mathrm{control}}_{it}$ indicates the set of control variables that may have an impact on the ULGUE. The constant term in the formula is $\alpha_{0}$, while $\alpha_{1}$, $\alpha_{2}$, $\gamma_{1}$ all represent the influence coefficients. $\mu_{i}$ indicates the urban fixed effect, and $\varepsilon_{\mathrm{it}}$ indicates a random error term.

#### 3.1.2. Mechanism Test Model

After examining the impact of growth targets management and regional competition on the ULGUE, this paper used the method of Cutler and Lleras-Muney (2010) [35] to build the following mechanism test model, and thereby identify the transmission mechanism of its impact.

First of all, the urban land green use efficiency ($ULGUE_{\mathrm{it}}$) regresses growth targets management ($gt_{it}$) and its regional competition ($w_{t}\cdot gt_{it}$) (as shown in formula (2)). Secondly, the mediation variable ($med_{it}$) regresses growth targets management ($gt_{it}$) and its regional competition ($w_{t}\cdot gt_{it}$) (as shown in formula (3)). Thirdly, urban land green use efficiency ($ULGUE_{\mathrm{it}}$) regresses growth targets management ($gt_{it}$) and its regional competition ($w_{t}\cdot gt_{it}$), combined with the mediator variables.

(2) $$ULGUE_{it}=\alpha_{0}+\alpha_{1}gt_{it}+\alpha_{2}w_{t}\cdot gt_{it}+\gamma_{1}control_{it}+\mu_{i}+\varepsilon_{it}$$

(3) $$med_{it}=\beta_{0}+\beta_{1}gt_{it}+\beta_{2}w_{t}\cdot gt_{it}+\gamma_{2}control_{it}+\mu_{i}+\varepsilon_{it}$$

(4) $$ULGUE_{it}=c_{0}+c_{1}gt_{it}+c_{2}w_{t}\cdot gt_{it}+c_{3}med_{it}+\gamma_{3}control_{it}+\mu_{i}+\varepsilon_{it}$$

In the following formulas, $med_{it}$ represents the mediator variable, including local investment attraction ($fid_{it}$), innovation input ($tec_{it}$), environmental regulation ($er_{it}$), and commercial activity ($ca_{it}$). $\alpha_{0}$, $\beta_{0}$, and $c_{0}$ represent the constant terms, while $\alpha_{1}$, $\alpha_{2}$, $\beta_{1}$, $\beta_{2}$, $c_{1}$, $c_{2}$, $c_{3}$, $\gamma_{1}$, $\gamma_{2}$, and $\gamma_{3}$ all represent the influence coefficients. When it comes to impact of growth targets management, $c_{1}$ represents the direct impact while $c_{2}$ represents the direct impact effect of the regional competition. When it comes to mediating effects, $\beta_{1}c_{3}$ represents the mediating effect for growth targets management, while $\beta_{2}c_{3}$ represents the mediating effect of the regional competition. The meaning of other variables is consistent with those in Formula (1).

A mediating effect exists for growth targets management ($gt_{it}$) if $\alpha_{1}$, $\beta_{1}$, and $c_{1}$ are simultaneously significant. A complete mediating effect exists if $c_{1}$ is not significant. If $\alpha_{1}$ is significant and $c_{1}<a_{1}$, there is a partial mediating effect that can be measured by $\beta_{1}c_{3}$. Similarly, a mediating effect exists for the regional competition of growth targets management ($w_{t}\cdot gt_{it}$) if $\alpha_{2}$, $\beta_{2}$, and $c_{2}$ are simultaneously significant. A complete mediating effect exists if $c_{2}$ is not significant. If $\alpha_{2}$ is significant and $c_{2}<\alpha_{2}$, there is a partial mediating effect that can be measured by $\beta_{2}c_{3}$.

### 3.2. Variable Selection

#### 3.2.1. Explained Variable the Urban Land Green Use Efficiency (ULGUE)

Scholars have had various understandings of the concept of ULGUE. Referring to the study of Pang et al., (2018) [36], this paper defined the ULGUE as a situation in which effective resources are invested in limited land to maximize economic, social, and environmental benefits by taking minimalization of environmental consumption as the principle under certain conditions. Based on the theoretical connotation of urban land green use, this paper constructed the evaluation index system of the ULGUE, measured the ULGUE of 257 prefecture-level cities from 2010 to 2017, and made a statistical analysis of the measurement results to provide data support for the empirical analysis of the later article.

(1) The Evaluation Index System for ULGUE

ULGUE is the core requirement for optimal allocation and effective use of land in contemporary social and economic development. It is also a crucial practice to implement the concept of “clear waters and green mountains are gold and silver mountains.” China’s economic development is gradually changing from an extensive model of pursuing rapid changes to the connotation mode of structural adjustment and environmental efficiency. This paper constructed an evaluation index system for the ULGUE (Table 1), selecting indicators from five dimensions: economy, environment, population, capital, and land.

This paper deals with several kinds of indicators. First, there are constant price GDP indicators from the economic dimension. Second, there are indicators of industrial soot, wastewater, and sulfur dioxide emissions from the environmental dimension. Finally, from the population dimension, there are indicators of the year-end unit employees and total year-end population. The indicator of capital stock and the indicators of built-up area and urban construction land were selected from the capital dimension and the land dimension, respectively.

When it comes to the input indicator, it is necessary to integrate land, economic, capital, and other inputs into the evaluation index system, since land is one of the input factors in the production process. Furthermore, since urban construction land and built-up areas are the primary sources of environmental pollution and damage, this study focused mainly on the indicators of built-up areas and urban construction land.

As for the output indicator, expected economic benefits and undesirable pollution are produced during land-use. For this paper, undesirable output was chosen based on the availability of data and considering that industry is the primary source of urban pollution. Therefore, undesirable output is the emission of industrial wastewater, industrial sulfur oxide and industrial powder dust. This indicates emission of urban wastewater, gas, solid waste, and other primary pollutants.

(2) The Measurement Method of ULGUE

The data envelopment analysis (DEA) model fails to perform an economic efficiency measurement containing undesirable output. To solve this problem, some scholars have proposed the widely used directional distance function. However, this method also has some defects. If there are input-output slack variables in the production system, the efficiency value will be too high. Tone (2001) [37] proposed the SBM model to compensate for this deficiency, putting slack variables into the target function in order to avoid errors caused by slack variables and angle selection, which has been widely applied by scholars to measure ULGUE. Therefore, this paper measured the ULGUE including undesirable output using the super efficiency SBM model based on the panel data of 257 prefecture-level cities from 2010 to 2017.

This paper treated each city as a decision-making unit (DMU). Let us assume that each DMU has m kinds of input ${x=(x}_{1},\cdots {,x}_{m})\in R_{+}^{m}$. Each DMU produces n kinds of expected output ${y=(y}_{1},\cdots {,y}_{n})\in R_{+}^{n}$ and k kinds of undesirable output ${b=(b}_{1},\cdots {,b}_{k})\in R_{+}^{k}$. In that case, the input and output values of the $j^{th}$ province (city, district) of phase t can be expressed as $\left(x^{j,t},y^{j,t},b^{j,t}\right)$, which means that the set of production possibilities for measuring land use efficiency can be constructed as:

(5) $$P^{t}(x^{t})=\left\{(y_{}^{t},b_{}^{t})|{\bar{x}}_{jm}^{t}\geq \sum_{j=1}^{J}\lambda_{j}^{t}x_{jm}^{t},{\bar{y}}_{jn}^{t}\leq \sum_{j=1}^{J}\lambda_{j}^{t}y_{jn}^{t},{\bar{b}}_{jk}^{t}\geq \sum_{j=1}^{J}\lambda_{j}^{t}b_{jk}^{t},\lambda_{j}^{t}\geq 0,\forall m,n,k\right\}$$

Based on the study of Tone (2001) [37], the super efficiency SBM model was constructed as:

(6) $$\rho^{*}=\min\frac{1-\frac{1}{m}\sum_{i=1}^{m}\frac{{\bar{x}}_{i}}{x_{i0}^{}}}{1+\frac{1}{n+k}(\sum_{r=1}^{n}\frac{{\bar{y}}_{r}^{}}{y_{r0}^{}}+\sum_{l=1}^{k}\frac{{\bar{b}}_{l}^{}}{b_{l0}^{}})}\;s.t.\left\{ \begin{array}{l} x_{0}=\sum_{j=1,\neq 0}^{J}\lambda_{j}^{}x+\bar{x},\\ y_{0}=\sum_{j=1,\neq 0}^{J}\lambda_{j}^{}y-\bar{y},\\ b_{0}=\sum_{j=1,\neq 0}^{J}\lambda_{j}^{}b+\bar{b},\\ \bar{x}\geq 0,\bar{y}\geq 0,\bar{b}\geq 0,\lambda_{j}^{}\geq 0 \end{array}\right.$$

In the formula, $\bar{x}$, $\bar{y}$ and $\bar{b}$ are the slack quantities of the input, expected output, and undesirable output, respectively. The weight factor $\lambda_{j}$ represents variable returns to scale if its sum is 1, or otherwise represents constant returns to scale. Considering the law of land investment output regarding return to scale, this paper selected variable returns to scale. In the target function, if $0\leq \rho^{*}\leq 1$, the larger the $\rho^{*}$, the higher the efficiency of the evaluation unit. Otherwise, it indicates that the evaluation unit is inefficient, and there is room for input-output improvement.

#### 3.2.2. Core Explanatory Variables

1. Growth targets management (gt). The economic growth target is the expected GDP growth set by the government after careful consideration, which carries strong authority and reflects the government’s commitment to a specific target task. Whether or not it is achieved has a substantial impact on the government and its relevant leaders. Therefore, the prefecture-level city governments realize the great importance of growth targets management, which has a great binding force and guiding nature on the government’s behavior during the following year. Growth targets management in this paper is defined as the management activities regarding economic growth targets carried out by local governments, measured by the original data of economic growth targets released by the Government Work Report, the Prefecture-level Statistical Yearbook, and the Five-year National Economic and Social Development Plan.
2. Regional competition ($w_{t}\cdot gt_{it}$). Regional competition, also known as the regional competition in growth targets management in this paper, refers to the spatial spillover effect of associated cities’ growth targets management on the ULGUE of local cities. It was measured by the interaction item of time-varying gravitational spatial weight matrix ($w_{t}$) and growth targets management (gt). The ordinary gravitational space weight matrix is static, with the implicit assumption that the economic connection between the phases is fixed between cities. However, this assumption does not fit the real economy. The economic links between cities change along with economic factors. Therefore, compared to the static weight matrix, the time-varying gravitational spatial weight matrix can better depict the dynamic characteristics of the regional competition evolution between the cities. The time-varying gravitational spatial weight matrix can also more accurately measure the spatial spillover effect of the growth targets management on the ULGUE. Therefore, drawing on the practice of Liu et al. (2015) [38], the paper depicts the construction of this matrix by using the modified gravity model. The specific model is as follows:
  (7) $$w_{ijt}=\{\begin{array}{c} \frac{gt_{it}}{gt_{it}+gt_{jt}}\times \frac{g_{it}g_{jt}}{d_{ij}^{2}},i\neq j\\ 0,i=j \end{array}$$

In the formula, $g_{it}$ and $g_{jt}$ indicate the constant-price GDP of city i and city j in period t, respectively. $d_{ij}$ represents the geographical distance between city i and city j. The time-varying gravitational spatial weight matrix ($w_{t}$) is the result obtained by standardizing each row after generating each element of the matrix ($w_{\mathrm{ij}t}$).

#### 3.2.3. Control Variables

Many factors affect the efficiency of land use. Considering that omitted variables may cause some endogenous errors, this paper included the following control variables.

1. Economic development level (lnpsg) was measured by the ratio of each prefecture level city’s GDP to the total population at the year end. On the one hand, improving economic development levels accelerates land circulation and promotes land-use efficiency. On the other hand, with the continuous improvement in economic development, the contradiction between supply and demand of land resources becomes more and more serious, which will not be conducive to the improvement of ULGUE. Therefore, this paper considered that economic development would affect ULGUE and brought it into the control variables for regulation.
2. Population density (hum) was measured by the natural logarithmic form of standardized treated population density. Population density has a relatively direct impact on the severity of environmental pollution. Excessive population density will increase environmental pollution, which harms the ULGUE.
3. Financial development level (fin) was measured by the ratio of the year-end loan balance of financial institutions to GDP. The improvement in financial development level will have an impact on the change in regional industrial structure. Upgrading industrial structures will promote effective use of land resources. Therefore, the impact of financial development on ULGUE needs to be further studied, and this paper managed it as a control variable.

#### 3.2.4. Mediator Variables

This paper included the following mediator variables:

1. Local investment attraction ($fid_{it}$), measured by the standardized ratio of actual utilized foreign capital in the current year to GDP;
2. Innovation inputs ($tec_{it}$), measured by the standardized ratio of local financial science expenses to financial expenditure;
3. Environmental regulation ($er_{it}$), measured by the method of Liu and Gong (2018) [39] based on industrial soot, exhaust and sulfur dioxide emissions;
4. Commercial activities ($ca_{it}$), measured by the natural logarithmic form of the highway passenger transportation volume.

### 3.3. Data Source

This paper used the panel data on 257 prefecture-level cities from 2011 to 2017 for the empirical analysis. The relevant data for growth targets management originated from the Government Work Report and the Prefecture-level Statistical Yearbook. Some of the missing data was obtained through the Five-year National Economic and Social Development Plan. The urban latitude and longitude coordinate data was gathered from the Baidu Map Open Platform. The data used to calculate the ULGUE was obtained from the China Urban Statistical Yearbook and the Statistical Yearbook of various cities over the years. The database referred to indicators such as emission of wastewater, industrial soot and sulfur dioxide, the year-end unit employees and the total year-end population, the built-up areas, the urban construction areas, the capital stock, etc. Due to the lack of corresponding statistics, the following indicators were obtained by calculation. The constant-price GDP is the conversion of the GDP calculated at the current price to the value calculated at a fixed period price (base period). Due to the elimination of price changes, comparing the constant-price GDP in two different periods can reflect actual change in activities of all permanent resident units in a country or region. The capital stock was calculated by the method of Liu et al., (2021) [40]. The remaining data were gathered from the China Urban Statistical Yearbook and the CSMAR database. Some of the missing data were complemented by the method of linear interpolation.

## 4. Results

### 4.1. Spatial and Temporal Distribution Analysis

#### 4.1.1. Urban Land Green Use Efficiency (ULGUE)

To further explore the spatial and temporal distribution of ULGUE in various cities in China, this paper divided the ULGUE of 257 prefecture-level cities into four grades from low to high. In order to draw the spatial distribution map, three analysis sections were selected—2011, 2014 and 2017. The ULGUE of extremely low-efficiency areas, low-efficiency areas, high-efficiency areas, and extremely high-efficiency areas are 0 to 0.25, 0.25 to 0.5, 0.5 to 0.75, and 0.75 to 1.0, respectively.

**Figure 2:**
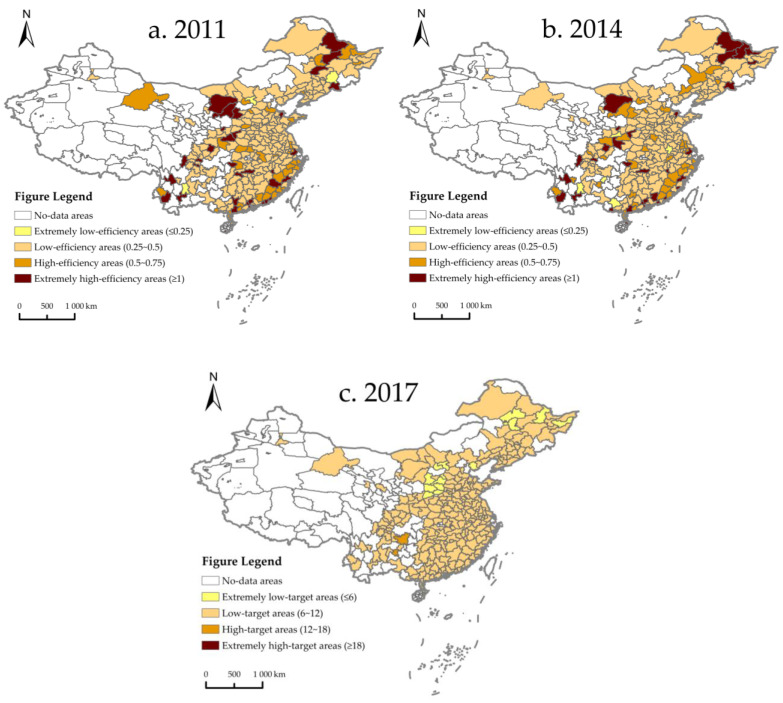
Spatial and temporal distribution of ULGUE in China. (**a**) Spatial distribution of ULGUE in 2011; (**b**) spatial distribution of ULGUE in 2014; (**c**) spatial distribution of ULGUE in 2017.

As shown in Figure 2, the overall green use efficiency of urban land in China is low, and most cities are low-efficiency areas. Comparing low and extremely low efficiency areas in 257 prefecture cities from 2011 to 2017, it can be observed that the share of low efficiency areas decreased from 70.03% to 67.70%, but it increased from 1.2% to 7.4% in extremely low efficiency areas. In 2011, only three cities had ULGUE of less than 0.25, namely Jilin and Datong in the central region and Kunming in the western region. However, in 2017, the number of cities with ULGUE below 0.25 increased significantly to 19, including eight in the eastern region: Shenyang, Anshan, Lianyungang, Yingkou, Linyi, Fushun, Nanning, and Baoding, seven in the central region: central Jilin, Qiqihar, Ganzhou, Datong, Huainan, Fuyang, and Changchun, and four in the western region: Lanzhou, Kunming, Xi’an and Guiyang (Table 2). This may imply that the current situation of ULGUE in Chinese cities is not optimistic.

#### 4.1.2. Economic Growth Target

**Figure 3:**
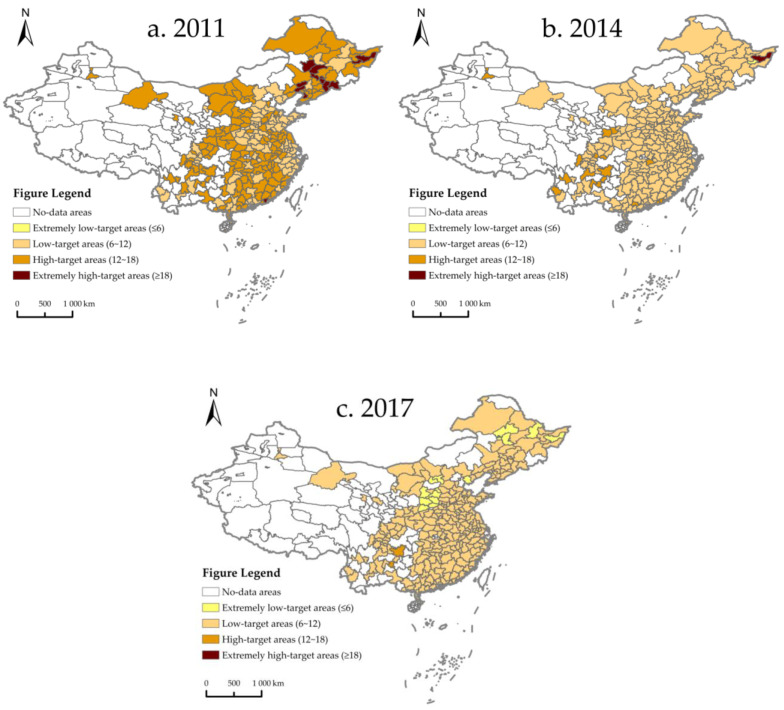
Spatial and temporal distribution of economic growth target in China. (**a**) Spatial distribution of economic growth target in 2011; (**b**) spatial distribution of economic growth target in 2014; (**c**) spatial distribution of economic growth target in 2017.

To further explore the spatial and temporal distribution of economic growth targets in various cities in China, this paper divided the economic growth target of 257 prefecture-level cities into four levels from low to high. Additionally, in order to draw the spatial distribution map, three analysis sections were selected—2011, 2014 and 2017. The economic growth targets of extremely low-target areas, low target areas, high target areas, and extremely high-target areas are 0 to 6, 6 to 12, 12 to 18, and 18 to 25, respectively. As shown in Figure 3, economic growth targets declined in most cities from 2011 to 2017. Looking at the period from 2011 to 2017, extremely high-target areas reduced from 13 to 1, and high-target areas increased sharply from 145 to 2. However, considering the same period of time, extremely low-target areas increased from 0 to 14, and low-target areas increased significantly from 99 to 241 (Table 3). This may imply that the economic growth targets set by Chinese cities show an overall trend of gradually slowing down.

### 4.2. Benchmark Regression Analysis

Based on the results of the Hausman test, the fixed effects model was used to perform regression analysis on 257 prefecture level cities. The results are shown in Models 1–4 of Table 4. Model 1 reports regression result of the ULGUE by growth targets management and regional competition without adding any control variables. Model 2 controlled the variable of economic development based on Model 1. Model 3 controlled the variable of population density based on Model 2 whereas Model 4 further controlled the variable of financial development based on Model 3.

In Model 1, both the regression coefficients of growth targets management and regional competition on the ULGUE were 0.005 and 0.097, respectively, significantly positive at 1% significance level. This could imply that growth targets management and regional competition significantly improve the ULGUE. After controlling the variable of economic development, the regression coefficients in Model 2 were 0.007 and 0.091 at 1% significance level, respectively. After further controlling the population density and financial development level, the regression coefficients of growth targets management in Model 3 and Model 4 were 0.008 and 0.009, and the regression coefficients of the regional competition were 0.11 and 0.111, respectively, all of which were significantly positive at the 1% level of significance. This may imply that the positive impact of growth targets management and its regional competition on the ULGUE remains.

Regarding control variables, the regression coefficient of economic development level and financial development level in Model 4 were 0.016 and 0.021, significantly positive at 10% and 5% significance level, respectively. This could imply that both economic and financial development contribute to improving the ULGUE. The regression coefficient of population density in Model 4 was −0.378, significantly negative at the 1% significance level. This may mean that population density has a significant inhibitory effect on the ULGUE.

### 4.3. Robustness Test

#### 4.3.1. Re-Measure the ULGUE

The above mentioned ULGUE incorporating undesirable outputs was calculated by the super efficiency slack-based model (SBM) which used the built-up area as the land indicator, and the total year-end population as the labor indicator. In the robustness test, the data for the land indicator and the labor indicator were replaced. The urban construction land area replaced the built-up area, and the year-end unit employees replaced the total year-end population. Two sets of ULGUE data were obtained using the super efficiency slack-based model (SBM). Model 5 represents the regression result after replacing the ULGUE that was calculated using the urban construction land area. The regression coefficients of growth targets management and its regional competition in Model 5 were 0.006 and 0.136, which remain significantly positive at the 1% significance level. Model 6 represents the regression result after replacing the ULGUE that was calculated using the year-end unit employees. The regression coefficients in Model 6 were 0.005 and 0.103, which also remain significantly positive at the 1% significance level. This may indicate that the research conclusions above are highly robust.

#### 4.3.2. Replace the Spatial Weight Matrix

According to the first law of geography, everything is related to everything else, but things that are close to each other are more related [41]. Therefore, this paper constructed a geospatial weight matrix (the reciprocal distance between the two cities) and re-estimated the benchmark model. The regression coefficient of growth targets management in Model 7 of Table 5 was 0.012, significantly positive at the 1% significance level. This may also imply that the abovementioned research conclusions have high robustness.

### 4.4. Mechanism Test

The abovementioned results showed that growth targets management has a strong positive impact on the ULGUE. How do growth targets management affect the improvement of the ULGUE? Next, this article will analyze this topic from four aspects: capital investment, technology investment, environmental regulation, and commercial trade. After identifying the mechanism, it was found that growth targets management and its regional competition will improve the ULGUE by promoting local investment attraction, increasing innovation inputs, optimizing environmental regulation, and strengthening commercial activities.

#### 4.4.1. Promoting Local Investment Attraction

Models 8–10 in Table 6 report the intermediary mechanism test results of local investment attraction. The regression coefficients of growth targets management and regional competition in Model 10 were smaller than in Model 8, significantly positive at 1% significance level. The result shows that growth targets management and regional competition can improve the ULGUE by promoting local investment attraction. The reason may be that local governments want to realize their economic targets, so they try promoting a large inflow of direct foreign investment by increasing land transfer and expanding fiscal expenditure. Further, it alleviates the financial constraints required for efficient and green land-use and ultimately improves its efficiency.

#### 4.4.2. Increasing Innovation Inputs

Models 11–13 in Table 7 report the intermediary mechanism test results of innovation inputs. The regression coefficients of growth targets management and its regional competition in Model 13 were smaller than in Model 11, significantly positive at 1% significance level. The result shows that growth targets management and regional competition can improve the ULGUE by increasing local governments’ investment in scientific and technological innovation. The reason may be that implementing critical tasks for innovative development is an important driving force for economic growth. Local government has to invest in technological innovation to achieve its economic growth targets. Adequate financial support provided by the government for scientific and technological innovation can effectively solve the financial problems faced by green land-use innovation, nurture the development of land green innovation technology, and ultimately promote the ULGUE.

#### 4.4.3. Optimizing Environmental Regulation

Models 14–16 in Table 8 report the intermediary mechanism test results of environmental regulation. The regression coefficients of growth targets management and regional competition in Model 16 are smaller than in Model 14, significant at 1%. This might indicate that growth targets management and regional competition can improve the ULGUE by optimizing environmental regulation. The reason may be that only by adhering to the green development concept can long-term economic development be achieved. A reasonable economic growth target and a proper regional competition will prompt local governments to focus on long-term economic development, implementing the concept of green development, and then optimizing local environmental regulation. The ULGUE can be improved by implementing environmental regulations since it can control bad behavior and reduce the internal driving force of pollutant discharge.

#### 4.4.4. Strengthening Commercial Activities

Models 17–19 in Table 9 report the intermediary mechanism test results of commercial activities. The regression coefficients of growth targets management and regional competition in Model 19 are smaller than in Model 17, significant at 1%. The results show that growth targets management and regional competition can improve the ULGUE by strengthening commercial activities. Since the commercial circulation industry can also significantly contribute to regional economic growth, setting economic growth targets will encourage local governments to vigorously develop this. Government will perhaps be encouraged also to invest in the development of commercial activities. Furthermore, the intensification of commercial activities can promote green development of cities to a certain extent, and thus improve the ULGUE.

### 4.5. Heterogeneity Analysis

#### 4.5.1. Heterogeneity Analysis of Regions

**Figure 4:**
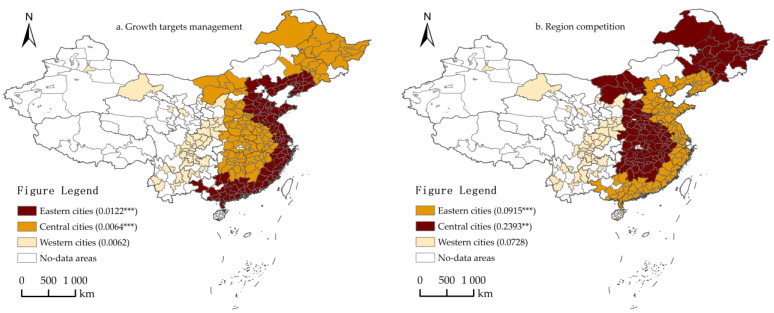
Heterogeneity analysis results (Regions). (**a**) The impact of growth targets management (gt) on ULUGE; (**b**) the impact of regional competition (w·gt) on ULUGE. *** and ** denote significance at the 0.01 and 0.05 levels, respectively. Except for the no-data area, the shade of the color represents the relative strength of the effect. The darker the color, the greater the relative strength of its effect.

To test the heterogeneity of the impact of growth targets management and its regional competition on the ULGUE in different regions, this paper divided all city samples into three sub-samples. According to the geographic areas, these sub-samples are eastern cities, central cities, and western cities. The results are shown in Figure 4 and Models 20–22 of Table 10. For one thing, the impact of growth targets management on the ULGUE was not significant in western cities but significantly positive at the 1% significance level in eastern cities and central cities. The regression coefficients were greater for eastern cities than for central cities. This might imply that growth targets management has no significant effect on the ULGUE of western cities, but has a significant effect on the ULGUE of eastern and central cities. It also has a more significant effect on eastern cities. In addition, the impact of regional competition on the ULGUE was not significant in western cities but significantly positive in eastern cities and central cities at 1% and 5% significance level, respectively. The regression coefficients were also greater for central cities than for eastern cities. This may imply that growth targets management has no significant effect on the ULGUE of western cities, but has a significant effect on the ULGUE of eastern and central cities and has a more significant effect on central cities.

#### 4.5.2. Heterogeneity Analysis of Administrative Levels

**Figure 5:**
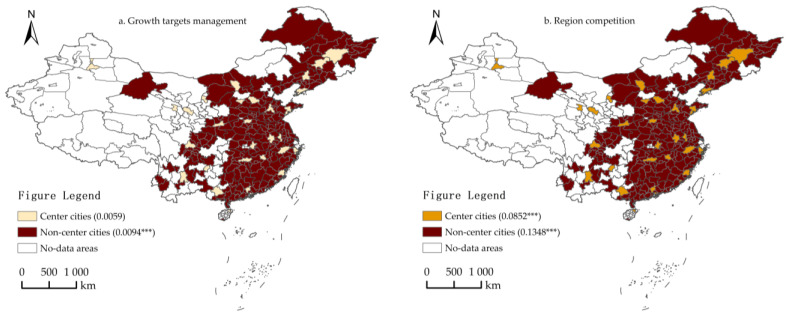
Heterogeneity analysis results (Administrative levels). (**a**) The impact of growth targets management (gt) on the ULUGE; (**b**) the impact of regional competition (w·gt) on the ULUGE. *** denotes significance at the 0.01 level. As above, the shade of the color represents the relative strength of the effect.

To test the heterogeneity that impact growth targets management and regional competition have on the ULGUE at different administrative levels, all city samples were divided into two sub-samples, central cities and non-central cities. The results are shown in Figure 5 and Models 23–24 of Table 11. On the one hand, the impact of growth targets management on the ULGUE was not significant in central cities, but significantly positive at the 1% significance level in non-central cities. The results show that growth targets management has no significant effect on the ULGUE of central cities, but has a significant effect on the ULGUE of non-central cities. On the other hand, the impact of the regional competition on ULGUE for both sub-samples was significantly positive at the 1% significance level, but the regression coefficients were greater for non-central cities than for central cities. The results show that regional competition has a significant effect on the ULGUE in cities at different administrative levels, but it has a more significant effect on non-central cities.

#### 4.5.3. Heterogeneity Analysis of Urban Agglomerations

**Figure 6:**
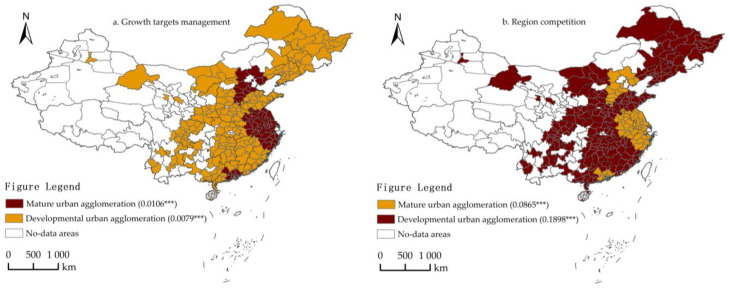
Heterogeneity analysis results (Urban agglomerations). (**a**) The impact of growth targets management (gt) on the ULUGE; (**b**) the impact of regional competition (w·gt) on the ULUGE. *** denotes significance at the 0.01 level. As above, the shade of the color represents the relative strength of the effect.

To test the heterogeneity of impact of growth targets management and regional competition on the ULGUE of different urban agglomerations, 18 of them were divided into two sub-samples for the purpose of analysis. Among them, mature urban agglomerations include the Beijing-Tianjin-Hebei urban agglomeration, Pearl River Delta urban agglomeration and Yangtze River Delta urban agglomeration. Results are shown in Figure 6 and Models 25–26 of Table 12. On the one hand, the impact of growth targets management on the ULGUE of the two sub-samples was significantly positive at 1% significance level, and the regression coefficients were greater for mature urban agglomerations than for developmental urban agglomerations. This might imply that the ULGUE of mature and developmental urban agglomerations are significantly affected by economic growth targets, and economic growth targets have a more significant effect on the ULGUE of mature urban agglomerations. On the other, the impact of regional competition on the ULGUE of the two sub-samples was significantly positive at 1%, and the regression coefficients were greater for developmental urban agglomerations than for mature urban agglomerations. This might imply that regional competition significantly affects the ULGUE of different urban agglomerations, and has a more significant effect on the ULGUE of developmental urban agglomerations.

## 5. Conclusions

Based on the 2010–2017 panel data of 257 prefecture-level cities, this paper measured the ULGUE containing undesired output by the super efficiency slack-based model (SBM). Then, it incorporated the regional competition of growth targets management into the analysis framework and characterized its dynamic evolution by constructing a time-varying gravitational spatial weight matrix. Additionally, this paper empirically tested the impact of the growth of targets management and regional competition on the ULGUE by the spatial self-lagging model. Moreover, it identified the mechanisms by which growth targets management and regional competition affect the ULGUE at the levels of capital investment, technology investment, environmental regulation and commercial trade. Finally, it analyzed the differentiation effect on the ULGUE from the perspective of multi-dimensional heterogeneity.

The main conclusions of this paper are as follows:

(1) growth targets management and regional competition have a significantly positive effect on ULGUE, and similar conclusions can still be drawn after re-measuring ULGUE and replacing spatial weight matrix, indicating that the research conclusion is highly robust;
(2) growth targets management and regional competition enhance the ULGUE by promoting local investment attraction, increasing innovation inputs, optimizing environmental regulation, and strengthening commercial activities;
(3) the effects of growth targets management and regional competition on cities in different regions, administrative levels and types of urban agglomerations differ; growth targets management plays a more significant role in promoting eastern and non-central cities and mature urban agglomerations, while regional competition plays a more significant role in central cities, non-central cities, and developmental urban agglomerations.

According to the research conclusions presented above and combined with China’s national conditions, the following suggestions are put forward:

(1) consider development as the priority and set aggressive economic growth targets to effectively promote the ULGUE; maintain appropriate local competition but also optimize the mechanism for competition in inter-regional growth targets management;
(2) further broaden the channels of regional investment, enrich the sources of local fiscal revenue, moderately improve the intensity of environmental regulation, and accelerate the development of electronic commerce technology and logistics technology, thus promoting local investment attraction, increasing innovation inputs, optimizing environmental regulation and strengthening commercial activities, all of which contribute to improving the ULGUE;
(3) encourage different cities to formulate economic growth targets that are in line with local conditions, considering the heterogeneity of regions, administrative levels and urban agglomerations; for eastern cities, non-central cities, or mature urban agglomerations, such as the non-central cities of Jiangsu Province and Zhejiang Province, which are also the cities of the Yangtze River Delta urban agglomeration, economic growth targets should continue to moderately rise and thus give full play to the positive role of growth targets management; for central cities, non-central cities, or developmental urban agglomerations, such as the non-central cities of Hunan Province, Hubei Province and Jiangxi Province, the focus should be on the synergy effect of growth targets management among related cities, promoting the flow of urban resource elements, and thus improving the ULGUE through inter-city cooperation and healthy competition.

## 6. Discussion

Considering the existing research, the advancement of the methodology in this paper can be reflected in two primary aspects: the measurement method of ULUGE and the method of exploring the spatial spillover effect.

Regarding the measurement method of ULUGE, this paper used the SBM model to calculate the ULGUE including undesirable outputs. In previous research, many scholars have widely used the Data envelopment analysis (DEA) model [42,43] and the directional distance function [44,45] to calculate land-use efficiency. However, the DEA model cannot measure economic efficiency containing undesirable output. The method of directional distance function also has a problem of too-high-efficiency values caused by the input-output slack variable. Therefore, this paper adopted the SBM model which helped us avoid the error caused by selection of slack variable and angle, by putting the slack variable into the target function.

Regarding the method of exploring the spatial spillover effect, this paper shows the application of the spatial self-lagging model to empirically investigate the impact of growth targets management and its regional competition on the ULGUE based on Vega et al. (2015). In previous studies, scholars usually used the spatial autoregression model, the spatial Durbin model, and the spatial self-lagging model to explore the spatial spillover effect between economic variables. However, the estimation processes of the first two models are cumbersome and complex. Therefore, this paper applied the spatial self-lagging model, a more straightforward method, to estimate the direct effects of growth targets management and regional competition. In conclusion, the results combined with the two above mentioned methods will have high practical value.

Although this paper specifically examines the impact of growth targets management and regional competition on ULUGE at both theoretical and empirical levels using 257 prefecture-level cities as the analytical sample, and draws some possibly valuable research conclusions and policy insights, there are still certain limitations that need to be further explored in future research.

(1) Broaden the research scope. This paper used the panel data on 257 prefecture-level cities for the empirical analysis, and the conclusions lack a certain degree of generalizability. Therefore, it is necessary to further broaden the scope of the analysis sample, and try to examine the direction and extent of the impact of economic growth targets on ULUGE in developed and developing countries, to test the generality of the findings of this paper, or dissect the reasons for the differences between developed and developing countries.
(2) Distinguish between different types of land. This paper only examined the effect of the regional competition of growth targets management on the green use efficiency of all types of land, instead of distinguishing between different types of land, such as agricultural land, construction land, etc. Thus, there is a need to analyze the similarities and differences of the effect of growth targets management and regional competition on the green use efficiency of different kinds of land, providing a more targeted reference basis for the government to enhance the green use efficiency of various types of land.
(3) Consider additional spatial factors. This paper only examined the effect of regional competition inb growth targets management on ULUGE, but in fact the spatial factors affecting ULUGE are not limited to this. For example, the regional strategic interactions of economic and financial development are also factors that could potentially influence this, which broadens our future research directions.

## Figures and Tables

**Table 1:** The evaluation index system for ULGUE.

First-Grade Indicators	Fundamental Indicators	Unit
Gross national product	Constant-price GDP	10,000 yuan
Environmental aspect	Industrial soot emissions	Ton
	Wastewater emissions	10,000 tons
	Sulfur dioxide emissions	Ton
Population situation	Year-end unit employees	10,000 people
	Total year-end population	10,000 people
Capital factor input	Capital stock	10,000 yuan
Urban land resources	Built-up area	Square kilometer
	Urban construction land area	Square kilometer

**Table 2:** Spatial and temporal distribution of ULGUE in China.

Number of Cities	Extremely Low-Efficiency Areas	Low-Efficiency Areas	High-Efficiency Areas	Extremely High-Efficiency Areas
2011	3	180	38	36
2014	5	179	38	35
2017	19	174	30	34

**Table 3:** Spatial and temporal distribution of economic growth target in China.

Number of Cities	Extremely Low-Target Areas	Low-Target Areas	High-Target Areas	Extremely High-Target Areas
2011	0	99	145	13
2014	1	238	17	1
2017	14	241	2	1

**Table 4:** Benchmark regression analysis.

Variable	Model 1	Model 2	Model 3	Model 4
gt	0.005 ***	0.007 ***	0.008 ***	0.009 ***
	(0.001)	(0.001)	(0.001)	(0.001)
w·gt	0.097 ***	0.091 ***	0.11 ***	0.111 ***
	(0.017)	(0.017)	(0.018)	(0.018)
ln psg		0.028 ***	0.017 **	0.016 *
		(0.008)	(0.009)	(0.009)
hum			−0.383 ***	−0.378 ***
			(0.095)	(0.096)
fin				0.021 **
				(0.01)

Note: ***, **, and * denote significance at the 0.01, 0.05, and 0.1 levels, respectively. Robust standard errors are in parentheses.

**Table 5:** Robustness test results.

Variable	Model 5	Model 6	Model 7
gt	0.006 ***	0.005 ***	0.012 ***
	(0.001)	(0.001)	(0.002)
w·gt	0.136 ***	0.103 ***	−0.006
	(0.028)	(0.025)	(0.004)
ln psg	0.049 ***	0.064 ***	0.03 ***
	(0.01)	(0.009)	(0.009)
hum	−0.506 ***	−0.37 ***	−0.14
	(0.1)	(0.096)	(0.097)
fin	0.007	0.01	0.021 **
	(0.011)	(0.01)	(0.01)

Note: *** and ** denote significance at the 0.01 and 0.05 levels, respectively. Robust standard errors are in parentheses.

**Table 6:** Mechanism test results (Local investment attraction).

Variable	Model 8	Model 9	Model 10
	LGUE	fdi	LGUE
gt	0.00873 ***	0.00130	0.00869 ***
	(0.001)	(0.001)	(0.001)
w·gt	0.11063 ***	0.02030	0.10811 **
	(0.018)	(0.016)	(0.018)
fdi			0.06195 **
			(0.031)
Control variable	Control	Control	Control

Note: *** and ** denote significance at the 0.01 and 0.05 levels, respectively. Robust standard errors are in parentheses.

**Table 7:** Mechanism test results (Innovation inputs).

Variable	Model 11	Model 12	Model 13
	LGUE	tec	LGUE
gt	0.00873 ***	0.00224 ***	0.00866 ***
	(0.001)	(0.001)	(0.001)
w·gt	0.11063 ***	0.02603	0.10797 ***
	(0.018)	(0.016)	(0.018)
tec			0.04550 *
			(0.027)
Control variable	Control	Control	Control

Note: *** and * denote significance at the 0.01 and 0.1 levels, respectively. Robust standard errors are in parentheses. Some of the data are retained with 5 decimal places to facilitate numerical comparisons.

**Table 8:** Mechanism test results (Environmental regulation).

Variable	Model 14	Model 15	Model 16
	LGUE	er	LGUE
gt	0.0087 ***	0.0177 ***	0.0081 ***
	(0.001)	(0.001)	(0.001)
w·gt	0.1106 ***	0.0031	0.1080 ***
	(0.018)	(0.002)	(0.018)
er			0.0368 ***
			(0.013)
Control variable	Control	Control	Control

Note: *** denotes significance at the 0.01 levels. Robust standard errors are in parentheses.

**Table 9:** Mechanism test results (Commercial activities).

Variable	Model 17	Model 18	Model 19
	LGUE	ca	LGUE
gt	0.0087 ***	0.0424 ***	0.0083 ***
	(0.001)	(0.006)	(0.001)
w·gt	0.1106 ***	0.3336 ***	0.1050 ***
	(0.018)	(0.103)	(0.018)
ca			0.0127 **
			(0.006)
Control variable	Control	Control	Control

Note: *** and ** denote significance at the 0.01 and 0.05 levels, respectively. Robust standard errors are in parentheses.

**Table 10:** Heterogeneity analysis results (Regions).

Variable	Eastern Cities	Central Cities	Western Cities
	Model 20	Model 21	Model 22
gt	0.0122 ***	0.0064 ***	0.0062
	(0.002)	(0.002)	(0.004)
w·gt	0.0915 ***	0.2393 **	0.0728
	(0.018)	(0.121)	(0.179)
Control variable	Control	Control	Control

Note: *** and ** denote significance at the 0.01 and 0.05 levels, respectively. Robust standard errors are in parentheses.

**Table 11:** Heterogeneity analysis results (Administrative levels).

Variable	Center Cities	Non-Center Cities
	Model 23	Model 24
gt	0.0059	0.0094 ***
	(0.005)	(0.001)
w·gt	0.0852 ***	0.1348 ***
	(0.022)	(0.029)
Control variable	Control	Control

Note: *** denotes significance at the 0.01 level. Robust standard errors are in parentheses.

**Table 12:** Heterogeneity analysis results (Urban agglomerations).

Variable	Mature Urban Agglomeration	Developmental Urban Agglomeration
	Model 25	Model 26
gt	0.0106 ***	0.0079 ***
	(0.004)	(0.001)
w·gt	0.0865 ***	0.1898 ***
	(0.014)	(0.065)
Control variable	Control	Control

Note: *** denotes significance at the 0.01 level. Robust standard errors are in parentheses.

## Data Availability

The data presented in this study are available on request from the first author.
